# Supplementary material for: Reduced Sulfur Diet Reshapes the Microbiome and Metabolome in Mild–Moderate Ulcerative Colitis
Source: Int J Mol Sci. 2025 May 11;26(10):4596. doi: 10.3390/ijms26104596 (PMC12111015; doi:10.3390/ijms26104596)
Supplement: Supplementary file 1 [file ijms-26-04596-s001.zip › ijms-3602843-supplementary.pdf]

Supplemental Files

Reduced Sulfur Diet Reshapes the Microbiome and Metabolome in Mild-Moderate Ulcerative Colitis

Authors:
Jiayu Ye,[1] Maitreyi Raman,[2] Lorian M Taylor,[2] Munazza Yousuf,[2] Remo Panaccione, [2] Christian Turbide,[2] Sidhartha R Sinha,[1] Natasha Haskey[3]

Table of Contents

Table S1. Demographic statistics 2
Table S2. Macronutrient and micronutrient content of the control and reduced sulfur diets at baseline and week 8. 3
Table S3. Diet indices of the control and reduced sulfur diet at baseline and week 8.......... 4
Table S4. Clinical biomarkers of the control and reduced sulfur diet at baseline and week 8 4
Table S5. Short-chain fatty acids of the control and reduced sulfur diet at baseline and week 8.......... 5
Table S6. Significantly changed metabolites at week 8 in the reduced sulfur group 6
Table S7. Significantly changed metabolites at week 8 in the control group.......... 6
Table S8. Taxa changes at week 8 in the RS group.......... 7
Table S9. Taxa changes at week 8 in the control group 7
Table S10. Significant Microbiome-Metabolite Correlations 8

Table S1. Demographic statistics

**Participant Characteristics**	**P value**
Sex, Female	0.0001
Age (years)	0.64
Body Mass Index (kg/m^2)	0.19
Fecal Calprotectin (mcg/g)	0.55

Table S2. Macronutrient and micronutrient content of the control and reduced sulfur diets at baseline and week 8.

Macronutrient Content Median (IQR)	Control (n=13)			Reduced Sulfur Diet (n=9)			P value[b]
	Baseline	Week 8	P value[a]	Baseline	Week 8	P value[a]	
Energy (kcal/day)	2430 (2103-2953)	1828 (1589-2290)	0.08	1941 (1398-2403)	1667 (1302-2224)	0.16	0.32
Protein (g/1000 kcal)	17 (13-23)	17 (15-20)	0.73	16 (11-21)	16 (14-20)	0.82	0.79
Fat (g/1000 kcal)	39 (26-40)	30 (25-36)	0.13	35 (28-49)	34 (32-45)	0.65	0.06
Carbohydrate (g/1000 kcal)	46 (34-54)	53 (37-59)	0.19	44 (31-56)	47 (36-53)	0.82	0.26
Fiber (g/1000 kcal)	8.4 (7.0-14)	9.5 (8.5-12)	0.68	9.4 (8.2-12)	9.4 (5.8-11)	0.07	0.27
Sugar (g/1000 kcal)	47 (26-66)	73 (34-77)	0.15	44 (25-69)	47 (36-60)	>0.99	0.16
**Micronutrient Content Median (IQR)**							
Calcium (mg/1000 kcal)	503 (323-621)	422 (277-577)	0.11	368 (335-671)	473 (295-662)	0.57	0.69
Iron (mg/1000 kcal)	6.1 (4.4-6.9)	6.0 (5.3-6.6)	0.84	6.6 (6.2-7.0)	6.8 (4.5-7.3)	0.57	0.61
Sodium (mg/1000 kcal)	1403 (1270-1734)	1418 (1144-1604)	0.59	1635 (1352-2143)	1711 (1380-1833)	0.82	0.11
Zinc (mg/1000 kcal)	4.7 (3.8-6.9)	5.0 (4.1-6.2)	0.79	5.8 (4.4-8.2)	5.5 (5.0-6.9)	0.20	0.32
Vitamin B12 (mcg/1000 kcal)	1.6 (1.0-2.6)	2.4 (1.5-2.7)	0.64	1.8 (1.3-2.8)	1.9 (1.2-3.4)	0.98	0.83
Choline (mg/1000 kcal)	164 (100-204)	156 (131-193)	0.46	159 (119-189)	193 118-277)	0.20	0.60

[a]Wilcoxon test; [b]Mann Whitney test; no differences seen at baseline between groups; p<0.05 considered significant.

Table S3. Diet indices of the control and reduced sulfur diet at baseline and week 8

Diet Characteristics Median (IQR)	Control (n=13)			Reduced Sulfur Diet (n=9)			P value[b]
	Baseline	Week 8	P value[a]	Baseline	Week 8	P value[a]	
Healthy Eating Index-2020[1]	74 (63-78)	70 (66-73)	0.83	71 (60-80)	67 (61-76)	0.65	> 0.99
Moderation Healthy Eating Index	24 (20-28)	27 (23-32)	0.21	21 (21-31)	27 (26-30)	0.29	0.88
Mediterranean Diet Adherence Score[2]	5 (4-6)	6 (4-7)	0.67	4 (3-6)	5 (5-6)	0.24	0.53

[a]Wilcoxon test; [b]Mann Whitney test; no differences seen at baseline between groups; p<0.05 considered significant.

[1] Shams-White MM, Pannucci TRE, Lerman JL, Herrick KA, Zimmer M, Meyers Mathieu K, et al. Healthy Eating Index-2020: Review and Update Process to Reflect the Dietary Guidelines for Americans, 2020-2025. J Acad Nutr Diet 2023;123. https://doi.org/10.1016/j.jand.2023.05.015.

[2] Papadaki, A.; Johnson, L.; Toumpakari, Z.; England, C.; Rai, M.; Toms, S.; Penfold, C.; Zazpe, I.; Martínez-González, M.A.; Feder, G. Validation of the English version of the 14-item mediterranean diet adherence screener of the PREDIMED study, in people at high cardiovascular risk in the UK. *Nutrients* **2018**, *10*, 138. https://doi.org/10.3390/nu10020138.

Table S4. Clinical biomarkers of the control and reduced sulfur diet at baseline and week 8

Clinical Biomarkers Median (IQR)	Control (n=13)			Reduced Sulfur Diet (n=9)			P value[b]
	Baseline	Week 8	p value[a]	Baseline	Week 8	p value[a]	
Fecal Calprotectin (mcg/g)	771 (96-1951) (n=12)	806 (84-1124) (n=12)	0.90	308 (30-1934) (n=7)	154 (54-295) (n=7)	0.94	0.33
Zonulin (ng/mL)	205 (101-351) (n=12)	123 (82-264) (n=12)	0.08	75 (245-396) (n=8)	262 (131-386) (n=8)	0.55	0.14
Lipopolysaccharide Binding Protein	11480 (9940-18840)	15800 (9810-23540)	0.41	13320 (9355-20378)	10510 (76930-11893)	0.20	0.06

[a]Wilcoxon test; [b]Mann Whitney test; no differences seen at baseline between groups; p<0.05 considered significant.

Table S5. Short chain fatty acids of the control and reduced sulfur diet at baseline and week 8

Short Chain Fatty Acids (µmol/g) median (IQR)	Control (n=13)			Reduced Sulfur Diet (n=8)			P value[b]
	Baseline	Week 8	p value[a]	Baseline	Week 8	p value[a]	
Total Short Chain Fatty Acids	32561 (26872-44750)	30690 (21253-41402)	0.10	27656 (19602-33699)	30214 (16463-34519)	0.64	0.13
Acetic Acid	0.53 (0.50-0.65)	0.59 (0.56-0.63) (n=12)	0.11	0.60 (0.54-0.63	0.57 (0.52-0.61)	0.22	0.48
Butyric Acid	0.19 (0.17-0.23)	0.17 (0.15-0.21) (n=12)	0.30	0.15 (0.12-0.16)	0.17 (0.13-0.20)	0.30	0.91
Propionic Acid	0.20 (0.01-0.03)	0.19 (0.14-0.21) (n=12)	0.52	0.19 (0.16-0.24)	0.20 (0.15-0.23)	0.58	0.47

[a]Wilcoxon test; [b]Mann Whitney test; no differences seen at baseline between groups; $p<0.05$ considered significant.

Fold Change in Short Chain Fatty Acids[a]	P value[b]
Total Short Chain Fatty Acids	0.18
Butyric Acid	0.06
Acetic Acid	0.45
Propionic Acid	0.50
Isobutyric Acid	0.62
Valeric Acid	0.03

[a]Calculated by change in absolute value (week 8/baseline)
[b]Mann Whitney test, $p < 0.05$ considered significant

Table S6. Significantly changed metabolites at week 8 in the reduced sulfur group

Compound Name	HMDB ID	Chemical Formula	Log2.FC.	P value	VIP	Correlation
4-Oxoproline	HMDB0304793	$C_5H_6NO_3$	7.14	0.047	2.08	0.50
Uric acid	HMDB0000289	$C_5H_4N_4O_3$	7.14	0.047	2.08	0.50
Methyluric acid	HMDB0001970	$C_6H_6N_4O_3$	2.31	0.036	2.18	0.53
Pyridoxolactone	HMDB0003454	$C_8H_7NO_3$	2.31	0.036	2.18	0.53
Noradrenochrome	HMDB0013030	$C_8H_7NO_3$	2.31	0.036	2.18	0.53
Methionine sulfoxide	HMDB0002005	$C_5H_{11}NO_3S$	4.68	0.005	2.73	0.66
Choline sulfate	HMDB0250194	$C_5H_{13}NO_4S$	4.68	0.005	2.73	0.66
2-Oxo-4-methylthiobutanoic acid	HMDB0001553	$C_5H_8O_3S$	4.68	0.005	2.73	0.66
N-Acetyl-L-glutamic acid	HMDB0001138	$C_7H_{11}NO_5$	1.73	0.024	2.32	0.56
Pyrocatechuic acid	HMDB0000397	$C_7H_6O_4$	1.73	0.024	2.32	0.56
Sodium thiosulfate	HMDB0303549	$Na_2O_3S_2$	-1.49	0.017	2.42	-0.58
3-Chlorotyrosine	HMDB0001885	$C_9H_{10}ClNO_3$	-0.92	0.022	2.35	-0.57
(2S)-2-[(2-Carboxy-2-oxoethyl)amino]pentanedioic acid	HMDB0257920	$C_8H_{11}NO_7$	2.33	0.034	2.20	0.53
QH(2)	HMDB0059661	$C_{14}H_{20}O_4$	1.36	0.040	2.14	0.52
Guanosine	HMDB0000133	$C_{10}H_{13}N_5O_5$	0.31	0.025	2.30	0.56
Hydroxydeoxyguanosine	HMDB0253263	$C_{10}H_{13}N_5O_5$	0.31	0.025	2.30	0.56
Glucose pyruvate	HMDB0252786	$C_9H_{14}O_9$	0.31	0.025	2.30	0.56
Indoleacetyl glutamine	HMDB0013240	$C_{15}H_{17}N_3O_4$	1.53	0.018	2.40	0.58
Tyrosyl-Lysine	HMDB0029110	$C_{15}H_{17}N_3O_4$	2.10	0.027	2.28	0.55
Pro-Pro-Pro	HMDB0256689	$C_{15}H_{17}N_3O_4$	2.10	0.027	2.28	0.55
24-Oxo-1alpha,23,25-trihydroxyvitamin D3	HMDB0060129	$C_{27}H_{42}O_5$	1.39	0.043	2.11	0.51
N-Eicosapentaenoyl Phenylalanine	HMDB0242077	$C_{29}H_{39}NO_3$	1.73	0.002	2.91	0.70

Table S7. Significantly changed metabolites at week 8 in the control group

Compound Name	HMDB ID	Chemistry Formula	Log2.FC.	pvalue	VIP	Correlation
1-(Hydroxymethyl)-5,5-dimethyl-2,4-imidazolidinedione	HMDB0031670	$C_6H_{10}N_2O_3$	-0.70	0.005	3.35	-0.38
Glycyl-Threonine	HMDB0028851	$C_6H_{12}N_2O_4$	-0.70	0.005	3.35	-0.38
Glutaminylhydroxyproline	HMDB0028798	$C_{10}H_{17}N_3O_5$	-0.79	0.044	2.45	-0.28
2H-1-Benzopyran-2-one, 7-(sulfooxy)-	HMDB0242135	$C_9H_6O_6S$	-0.55	0.050	2.39	-0.27
MG(10:0/0:0/0:0)	HMDB0072866	$C_{13}H_{26}O_4$	0.60	0.050	2.39	0.27
2-Ethylidene-1,5-dimethyl-3,3-diphenylpyrrolidine	HMDB0041803	$C_{20}H_{23}N$	0.56	0.038	2.52	0.29
Oxohexadecanoic acid	HMDB0010733	$C_{16}H_{30}O_3$	-0.64	0.047	2.42	-0.28
tryptophan glutamate	HMDB0259308	$C_{16}H_{19}N_3O_6$	1.58	0.013	3.00	0.34
Glucose lactate glutamate	HMDB0252780	$C_{14}H_{23}NO_{13}$	0.37	0.044	2.45	0.28

Table S8. Taxa changes at week 8 in the RS group

Taxon	Coefficient	P value
Eggerthella_lenta	-1.87	0.010
Asaccharobacter_celatus	-1.20	0.006
Adlercreutzia_equolifaciens	-1.32	0.019
Enterorhabdus_caecimuris	-0.97	0.017
Eubacterium_ramulus	-1.87	0.071
Alistipes_finegoldii	1.14	0.079
Collinsella_stercoris	0.78	0.086
*Actinomyces_sp_*HMSC035G02	1.21	0.080
Romboutsia_ilealis	-2.01	0.040

Table S9. Taxa changes at week 8 in the control group

Taxon	Coefficient	P value
Dorea_formicigenerans	0.40	0.099
Dorea_longicatena	0.30	0.063
Ruminococcus_torques	0.62	0.086
Romboutsia_ilealis	-1.23	0.086
Intestinibacter_bartlettii	-1.96	0.046

Table S10. Significant Microbiome-Metabolite Correlations

Pair	Correlation	P value
Sodium thiosulfate -- 3-Chlorotyrosine	0.65	<0.001
Pro-Pro-Pro -- N-Eicosapentaenoyl-Phenylalanine	0.64	0.007
Tyrosyl-Lysine -- N-Eicosapentaenoyl-Phenylalanine	0.64	0.007
Adlercreutzia_equolifaciens -- Asaccharobacter_celatus	0.68	0.013
Adlercreutzia_equolifaciens -- Eubacterium_ramulus	-0.67	0.015
2-Oxo-4-methylthiobutanoic acid -- 3-Chlorotyrosine	-0.66	0.016
Choline sulfate -- 3-Chlorotyrosine	-0.66	0.016
Methionine sulfoxide -- 3-Chlorotyrosine	-0.66	0.016
Sodium thiosulfate -- QH(2)	-0.64	0.041
Asaccharobacter_celatus -- *Eubacterium_ramulus*	-0.62	0.042
Eggerthella_lenta -- Methyluric acid	-0.77	0.043
Eggerthella_lenta -- Noradrenochrome	-0.77	0.043
Eggerthella_lenta -- Pyridoxolactone	-0.77	0.043
Eggerthella_lenta -- Glucose pyruvate	-0.67	0.045
Eggerthella_lenta -- Guanosine	-0.67	0.045
Eggerthella_lenta -- Hydroxydeoxyguanosine	-0.67	0.045
QH(2) -- Indoleacetyl.glutamine	0.84	<0.001
Collinsella_stercoris -- Methyluric acid	0.70	<0.001
Collinsella_stercoris -- Noradrenochrome	0.70	<0.001
Collinsella_stercoris -- Pyridoxolactone	0.70	<0.001
